# Using scenario tree modelling for targeted herd sampling to substantiate freedom from disease

**DOI:** 10.1186/1746-6148-7-49

**Published:** 2011-08-16

**Authors:** Sarah Blickenstorfer, Heinzpeter Schwermer, Monika Engels, Martin Reist, Marcus G Doherr, Daniela C Hadorn

**Affiliations:** 1 Veterinary Public Health Institute, Vetsuisse Faculty, University of Berne, Switzerland; 2 Swiss Federal Veterinary Office, Berne, Switzerland; 3 Institute of Virology, Vetsuisse Faculty, University of Zurich, Switzerland

## Abstract

**Background:**

In order to optimise the cost-effectiveness of active surveillance to substantiate freedom from disease, a new approach using targeted sampling of farms was developed and applied on the example of infectious bovine rhinotracheitis (IBR) and enzootic bovine leucosis (EBL) in Switzerland. Relevant risk factors (RF) for the introduction of IBR and EBL into Swiss cattle farms were identified and their relative risks defined based on literature review and expert opinions. A quantitative model based on the scenario tree method was subsequently used to calculate the required sample size of a targeted sampling approach (TS) for a given sensitivity. We compared the sample size with that of a stratified random sample (sRS) with regard to efficiency.

**Results:**

The required sample sizes to substantiate disease freedom were 1,241 farms for IBR and 1,750 farms for EBL to detect 0.2% herd prevalence with 99% sensitivity. Using conventional sRS, the required sample sizes were 2,259 farms for IBR and 2,243 for EBL. Considering the additional administrative expenses required for the planning of TS, the risk-based approach was still more cost-effective than a sRS (40% reduction on the full survey costs for IBR and 8% for EBL) due to the considerable reduction in sample size.

**Conclusions:**

As the model depends on RF selected through literature review and was parameterised with values estimated by experts, it is subject to some degree of uncertainty. Nevertheless, this approach provides the veterinary authorities with a promising tool for future cost-effective sampling designs.

## Background

Documented freedom from disease is the basis for international free trade of animals and animal products. In Switzerland, annual serological surveys are conducted to substantiate freedom from infectious bovine rhinotracheitis (IBR), enzootic bovine leucosis (EBL), *Brucella melitensis*, Aujeszky's disease and porcine reproductive and respiratory syndrome (PRRS). Switzerland is free of IBR and EBL since 1994. Therefore, a very low prevalence is considered for sample size calculation and, in consequence, a large sample size is required to demonstrate freedom from disease [1]. Thus, this active surveillance approach is costly and personnel-intensive. The development of cost-effective tools for animal disease surveillance is therefore of high interest to scientists and decision-makers in the field of veterinary public health.

One approach to increase the efficiency of active surveillance is targeting high-risk strata in the animal population, termed risk-based surveillance, or in the context of this manuscript called targeted sampling (*TS*). It is based on the identification and utilisation of specific, scientifically documented quantitative risk factors for occurrence of the respective diseases [2]. In conventional approaches to document disease freedom, a sample of farms is selected randomly from a central database. Randomness is necessary to ensure representativeness for induction on the population. Random sampling can be done in strata without violating the assumption of representativeness. However, random sampling does not take into account uneven distribution of disease risk. Thus, it is the best choice only in absence of information on the distribution of disease risk. When such information is available, this can be used to formulate risk strata. Consequently, testing high-risk strata offers a potential of detecting disease with a higher probability or a smaller sample size compared to testing non high-risk strata [2].

The aims of our study were to evaluate the performance and cost-effectiveness of a *TS *approach compared with conventional stratified random sampling (*sRS*) using stochastic scenario tree modelling [1,3]. Our study diseases for this task were IBR and EBL in Swiss cattle, the freedom of which we wanted to demonstrate at a maximum herd prevalence of 0.2% with an overall sensitivity of 99% [4]. Within the scope of this paper, the level of confidence yielded by a surveillance system is referred to as its sensitivity.

## Methods

### Evaluation of risk factors for IBR and EBL

The first step of the study consisted in identifying relevant risk factors (RF) for disease occurrence on individual cattle farms. A literature review on the epidemiology of IBR and EBL was conducted with a focus on specific RF for the diseases and their relevance for the Swiss cattle population, given that the population is considered to be free of these diseases. Lists of RF were generated and discussed with national experts within the field resulting in a final, strongly condensed selection of RF for both diseases (Table 1). As all cattle farms are registered in the animal movement database (TVD), the chosen RF could be allocated to the affected farms by means of the TVD and the geographical software ArcGis 9, ArcMap Version 9.2 (ESRI Inc.) providing us with an Excel list (Microsoft Corporation 2007) of all 52,176 Swiss cattle farms and their corresponding RF for IBR and EBL for the year 2008.

**Table 1:** Risk factors for the introduction of IBR and EBL into Swiss cattle farms

Risk factor (RF)	Farms exposed to RF	Definition of the risk involved
***IBR***		

**Animal contacts (*AC*)**	All farms which send their cattle, or part of it, to summer pastures (inside the country or across the border) and/or let their bovines participate in cattle shows	Physical contacts with potentially infected bovines from other farms

**Higher-than-average animal movements on farm (*AM*)**	All farms having more cattle entries on farm per year than the yearly median value for their herd size category	Farms which purchase many bovines from outside have a higher risk of getting an IBR-positive animal into their herd than farms which do not purchase any cattle

**Farm close to the border with another country (*FcB*)**	All cattle farms situated up to 5 km from the Swiss border and 500 m at most from a larger road (in this zone)	Uncontrolled contacts between potentially infected animals; airborne transmission of pathogens; veterinarians from neighbouring countries treating cattle (having contact with potentially IBR-infected animals); facilitated illegal importation of bovines

**High density of cattle farms in the vicinity (*hDH*)**	All farms that have many (in our case >21) neighbouring farms within a radius of 1 km around their farm	Uncontrolled contacts between animals (over fences), or between animals and persons (neighbouring families, visitors...)

**Importation of cattle (*IC*)**	All farms having imported cattle in their herds	Even though cattle destined for importation must originate from IBR-free herds, or, in the case of non-IBR-free countries, have to be tested for IBR, an introduction of the disease through cattle importation can never be excluded

***EBL***		

**Higher-than-average animal movements on farm (*AM*)**	All farms that have more cattle entries on farm per year than the yearly median value for their herd size category	Farms which purchase many bovines from outside, have a higher risk of getting an EBL-positive animal in their herd than farms which do not purchase any cattle

**Importation of cattle (*IC*)**	All farms having imported cattle in their herds	Even though cattle destined for importation must originate from EBL-free herds, or, in the case of non-EBL-free countries, have to be tested for EBL, an introduction of the disease through cattle importation can never be excluded

**Summer pasture with animals from other herds (*SP*)**	All farms which send their cattle, or part of it, on summer pastures (inside the country or across the border)	This risk factor implicates *lengthy *physical contacts between animals from different herds and therefore makes a transmission of EBL from one bovine to another possible; cattle are exposed to biting and stinging insects in the summer season (→ transmission of infected lymphocytes)

To allow quantitative comparison of information content or gain of RFs - either single or combined - these had to be parameterised. To allocate values to the selected RF, we chose a modified Delphi approach for the gathering of expert opinion [5]. An electronic questionnaire, seeking estimations on the minimum, the most likely and the maximum values of relative risks (RR) for the selected RF (i.e. the expert-based change in disease risk compared to a baseline RF level) was sent to 15 experts in the field of veterinary epidemiology, veterinary virology and veterinary public health. We formulated the questions without giving a desired range for the RR values. Thus, the experts were boundless free to name their estimates. The same questionnaire, supplemented with the median values of all RR estimates from the first round was sent to the experts a second time, offering them the possibility to either adjust their estimates for the RR or to confirm their previous values. The final results considered for the parameterisation of the RF were the median values of all estimates from the second questioning round.

### Adaptation and development of the scenario tree model

**Figure 1:**
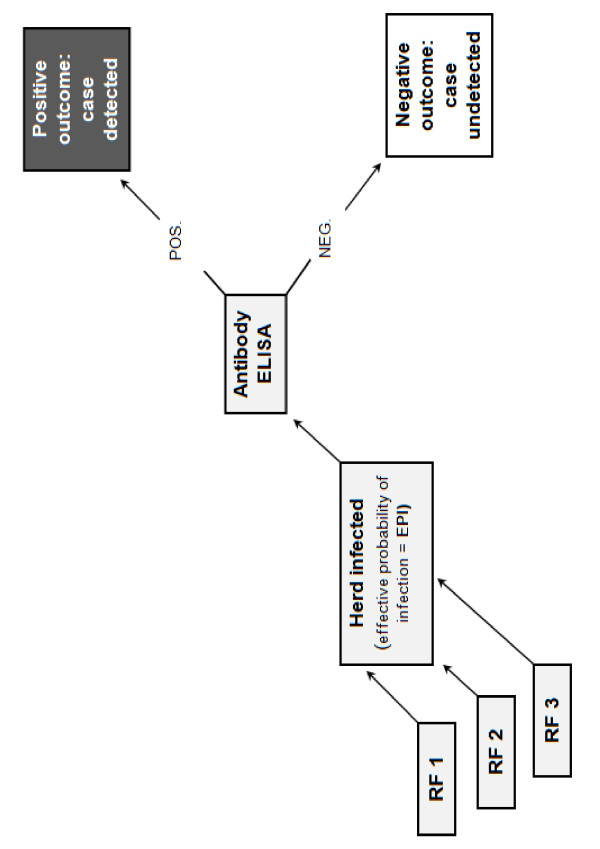
**Conceptual schematic of the scenario tree for the annual serological survey to demonstrate freedom from EBL in Switzerland**. For IBR, the same procedure applies, but with 5 instead of 3 risk factors (RF).

A scenario tree models the process of disease detection through a surveillance system component (SSC), tracing the probabilities that a single unit (eg. farm) will yield either a positive or a negative outcome. The tree includes all factors affecting the probability of infection or detection of a surveillance unit (Figure 1). Scenario tree modelling is used to calculate the sensitivity of a SSC for a given design prevalence and sample size [3].

The scenario tree models described in literature so far are mostly used to calculate the sensitivity of a SSC for a given sample size and design prevalence [1,3]. In our use of the scenario tree method, we also aimed at calculating a sample size to demonstrate disease freedom for a given sensitivity and at a given prevalence. Hence, the SSC evaluated in our study was the annual serological survey for IBR and EBL. We also wanted to determine the risk factor combinations (eq. risk strata) that yielded the highest information gain, so as to choose as many farms as possible from these high-risk strata and, in consequence, keep the sample size minimal. We parameterised two models (one for IBR, one for EBL) using the corresponding data for the respective diseases and modelled two sampling scenarios, one hypothetical and one practical, for each disease.

#### Input parameters

In the scenario tree model, we used a design prevalence at the herd level (P*H) of 0.2% for both IBR and EBL (Table 2). We defined the proportions (*Pr_RF_*) of the selected risk factors for Swiss cattle farms as well as their RR (*RR_RF_*) (Table 2) for the calculation of the adjusted risks and the effective probabilities of infection (EPI) for each combination of risk factors and hence, every single farm [1,3]. The medians of the minimum, the most likely and the maximum values for the RR determined through expert opinion were modelled as pert distributions in @Risk 5.0 (Palisade Corporation) and run with 1000 iterations. The test sensitivities for the antibody-ELISAs (CHEKIT^® ^Trachitest Serum, IDEXX Laboratories and CHEKIT^® ^Leucose Serum, IDEXX Laboratories) used for the annual serological surveys in Switzerland were set at 99.3% for IBR and 99.9% for EBL. These values were obtained from the Swiss reference laboratories for IBR and EBL (Table 2). All animals per herd are tested and a herd is already classified positive if only one single sample shows a positive reaction. Therefore, the values for the herd sensitivities are equal to the values of the diagnostic test (= single unit) sensitivities. The specificity of both ELISA-tests was set at 100%, since Switzerland is free of both diseases and every positive test result would consistently be retested with additional diagnostic tests until confirmed positive or negative. Therefore, the model does not account for false positive results.

**Table 2:** Input parameters used in the scenario tree model to substantiate freedom from IBR and EBL in Switzerland

Description of input parameter	Value	Source
Herd-level design prevalence for disease freedom from IBR and EBL (P*H)	0.002	OIE Animal Health Code 2010

**Proportions of risk factors for IBR in the cattle farm population (Pr_RF_)**		

Proportion of "animal contacts" (Pr_AC_)	0.401	TVD^1^
Proportion of "animal movements" (Pr_AM_)	0.286	TVD^1^
Proportion of "farm close to border" (Pr_FcB_)	0.100	TVD^1^
Proportion of "high density of herds" (Pr_hDH_)	0.123	TVD^1^
Proportion of "importation of cattle" (Pr_IC_)	0.002	TVD^1^

**Proportions of risk factors for EBL in the cattle farm population (Pr_RF_)**		

Proportion of "animal movements" (Pr_AM_)	0.286	TVD^1^
Proportion of "importation of cattle" (Pr_IC_)	0.002	TVD^1^
Proportion of "common summer pasture" (Pr_SP_)	0.398	TVD^1^

**Relative risks (RR_RF_) of risk factors for IBR**		

RR of "animal contacts" (RR_AC_)	RiskPert(2; 4; 6)	Expert opinion^2^
RR of "animal movements" (RR_AM_)	RiskPert(2; 4; 6)	Expert opinion^2^
RR of "farm close to border" (RR_FcB_)	RiskPert(2; 4; 6)	Expert opinion^2^
RR of "high density of herds" (RR_hDH_)	RiskPert(1; 2; 3)	Expert opinion^2^
RR of "importation of cattle" (RR_IC_)	RiskPert(2; 4; 6)	Expert opinion^2^

**Relative risks (RR_RF_) of risk factors for EBL**		

RR of "animal movements" (RR_AM_)	RiskPert(1; 2; 3)	Expert opinion^2^
RR of "importation of cattle" (RR_IC_)	RiskPert(1.5; 4; 5)	Expert opinion^2^
RR of "common summer pasture" (RR_SP_)	RiskPert(1; 1.5; 3)	Expert opinion^2^

**ELISA test-sensitivities for herd serology (T_SensSH_)**		

T_SensSH _of IBR-Antibody-ELISA (CHEKIT^® ^Trachitest Serum, IDEXX Laboratories)	0.993	Swiss Reference Laboratory for IBR^3^
T_SensSH _of EBL-Antibody-ELISA (CHEKIT^® ^Leucose Serum, IDEXX Laboratories)	0.999	Swiss Reference Laboratory for EBL^4^

^1^Swiss Animal Movement Database (2008)

^2^Modified Delphi approach

^3^Institute of Virology, University of Zurich

^4^Institute of Veterinary Virology, University of Berne

#### Model Outputs

With the adapted scenario tree model, we were able to calculate the sensitivity of a certain targeted risk stratum using the following equation described by Martin et al. [3]:(1)

where *CSe *(component sensitivity) is the sensitivity of a certain risk stratum, *SUSe *(system unit sensitivity) corresponds to the average probability that one randomly selected farm out of a certain risk stratum will yield a positive outcome, given that the country is infected at P*H. *SUSe *is calculated by summing up the limb probabilities for all limbs with positive outcomes in the scenario tree model.

The sample size *n *for conducting a random sample or a targeted sample in a certain risk stratum required to reach a given sensitivity *CSe *can be calculated by solving equation (1) for *n*:(2)

As we were interested in calculating a sample size containing farms from different, especially high-risk strata, we needed to combine the *CSe *of several targeted risk strata to an overall *SSe *(system sensitivity) for all selected risk strata. This was done using the following equation described by Martin et al. [3]:(3)

where *J *denotes the number of risk strata considered and *CSe_j _*corresponds to the sensitivity for the *j-*th risk stratum.

##### (a) Using exclusive targeted sampling

For both diseases, a theoretical scenario involving solely *TS *in the highest risk stratum was modelled. In this scenario, only farms possessing all RF for the respective diseases, meaning that they were classified in the risk stratum with the greatest information gain, were considered for sampling. However, this implies that enough farms comprising each of the studied RF must exist, which, in reality was not the case. Exclusive *TS *in a practical sampling scenario is also possible. However, this means that farms from several risk strata would have to be considered for testing. The reduction in sample size would then be smaller than in the hypothetical scenario mentioned above, where only farms from the highest risk stratum are considered.

##### (b) Combining targeted and random sampling in one sampling scheme

In order to make use of the information gain of *TS *but without compromising the representativeness of the survey with a very small targeted sample size, we combined *TS *with a baseline stratified random sample (*bsRS*). This combined approach *cTS&bsRS *is conducted as follows: First, the sensitivity of a *bsRS *has to be determined. In our example, we decided to conduct a *bsRS *with a sensitivity of 90%. The sample size for the *bsRS *was calculated using equation (2) and instead of targeting a certain risk stratum, we ran the scenario tree model distributing the *Pr_RF _*as they appear in the population, so as to obtain a sample chosen randomly out of all risk strata.

**Figure 2:**
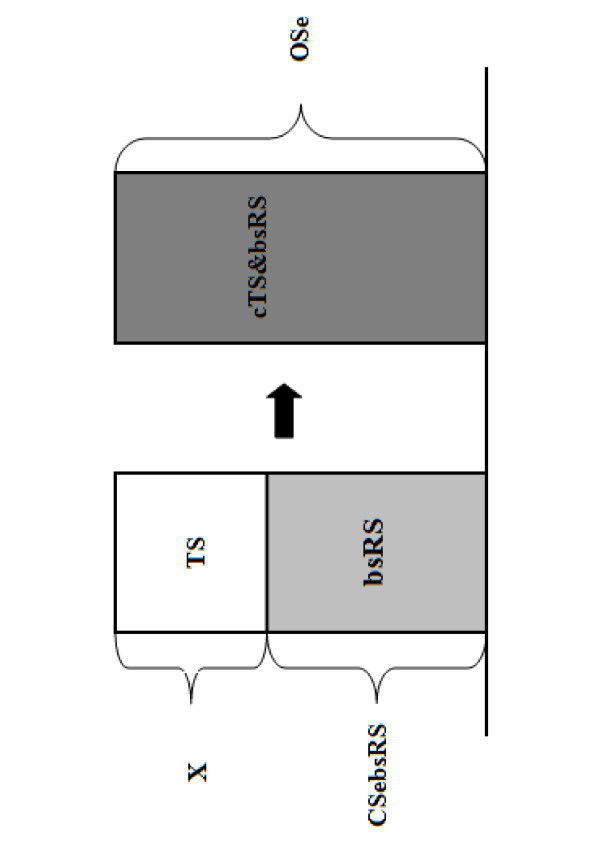
**Conceptual schematic representing the process of combining targeted sampling with baseline random sampling to substantiate freedom from disease**. ***TS ***is the targeted sampling component, ***bsRS ***denotes the baseline stratified random sampling component with the sensitivity ***CSebsRS***, ***cTS&bsRS ***is the combination of targeted and random sampling, ***OSe ***is the required overall sensitivity to demonstrate freedom from disease and ***X ***represents the sensitivity of the *TS *component, the value of which can be calculated using eq. (4).

In order to reach the overall sensitivity (*OSe*) of 99% for the documentation of freedom from disease, the required *SSe *for the *TS *component can be calculated using the following formula, modified from Hadorn et al. [6]:(4)

where *x *is the required *SSe *for the *TS *component, *OSe *the required overall sensitivity of *cTS&bsRS *and *CSebsRS *the sensitivity of the baseline random sample (Figure 2).

### Analysis of cost-effectiveness

To compare the cost-effectiveness of the *cTS&bsRS *approach with that of *sRS*, we identified the differences in each step of the planning and implementation of the annual serological surveillance programme for the two methods. We then determined the costs linked to each step of the programme, based on already available data from the Swiss Federal Veterinary Office (FVO) (unpublished data: Sonia Menéndez, "Costs of surveillance systems (2008)", Monitoring Department, Federal Veterinary Office) and compared the resulting full survey costs for both approaches.

In Switzerland, all cattle over two years of age on selected survey farms are tested for IBR and EBL [7]. In order to get an estimate of cost for budgeting, we calculated the net costs of the samples (material and analysis) for the average number of individual animals to be tested per farm at 20 for randomly selected farms (which corresponds to the long-time average [7]), and in targeted selected farms at 30 animals per farm due to the larger average herd sizes in "risk farms" (corresponding to an average of 30 animals per farm, as deduced from our data). The detailed effective costs have to be calculated at the end of the survey.

## Results

### Evaluation of risk factors for IBR and EBL

As a result of the literature review and expert opinion survey, we identified the five following relevant RF for the introduction of IBR into Swiss cattle farms together with the corresponding sets of minimum, most likely and maximum values for their RR (in brackets): *animal contacts (2/4/6)*, *over-average animal movements (2/4/6)*, *farm close to the border (2/4/6)*, *importation of cattle (2/4/6) *and *high density of herds in the vicinity (1/2/3) *(Table 1) [8-27]. With five RF and two possible outcomes (yes/no) each, we could generate 32 (= 2^5^) possible different combinations of RF, meaning that we had 32 risk strata for IBR available in the scenario tree model. As a result, we could assert that ~ 40% (20,870) of all cattle farms had no RF for IBR, whereas only one farm (~ 0.002%) had all five RF for the disease.

For EBL, the following three RF were determined, together with the corresponding sets of minimum, most likely and maximum values for their RR (in brackets): *importation of cattle (1.5/4/5), over- average animal movements (1/2/3)*, and *summer pasture with other herds (1/1.5/3) *(Table 1) [28-45]. With three risk factors and two possible outcomes, we could generate 8 (= 2^3^) different risk strata for the EBL model. ~51.5% (26,870) of all cattle farms had no RF for EBL, while ~ 0.07% (40) of the farms had all three RF applying to them.

### Adaptation and development of the scenario tree model

#### (a) Using exclusive targeted sampling

The theoretical sample sizes of the scenario involving solely *TS *in the highest risk stratum ranged from 21 to 58 farms (yielding a 99% sensitivity on the 95%- and 5%-percentile, respectively, on the distribution for *CSe*) for IBR and 208 to 486 farms (95%- and 5%-percentile, respectively) for EBL.

#### (b) Combining targeted and random sampling in one sampling scheme

**Figure 3:**
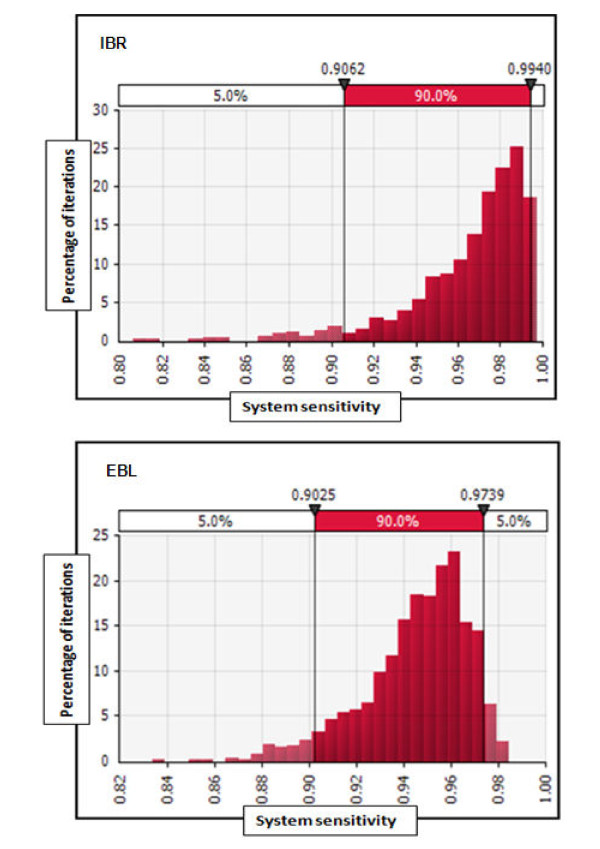
**Distribution of the system sensitivity (*SSe*) of the targeted sampling component (*TS*) for IBR (n = 83 farms) and EBL (n = 600 farms)**.

The sample sizes for *bsRS *resulted in 1,158 and 1,150 farms for IBR and EBL, respectively. The difference of 8 farms between IBR and EBL is explained by the difference in the test sensitivity for the two diseases (Table 2). The *SSe *required for the *TS *component calculated with equation 4 accounted for 90% in order to reach an *OSe *of 99% (Figure 2). The sample sizes for the *TS *component selected out of the highest risk strata for the respective diseases consisted of 83 farms for IBR and 600 farms for EBL in order to yield 90% sensitivity on the 5%-percentile. We used the results on the 5%-percentile as a conservative approach in defining the sample sizes for the *TS *component (Figure 3).

For sampling, the farms, as they actually existed in reality, were successively selected out of the highest risk strata until the required *SSe *was reached. For IBR, this resulted in: the only farm out of the highest risk stratum with all RF present; all of the two farms out of the stratum with the RF *AC, AM, FcB *and *IC *present and 80 farms out of 125 actually available farms in the stratum with the RF *AC, AM, FcB*, and *hDH *present. For EBL this resulted in: all of the 40 farms out of the highest risk stratum with all RF present; all of the 18 farms out of the risk stratum with RF *AM *and *IC*; all of the 8 farms out of the risk stratum with RF *SP *and *IC*; all of the 34 farms out of the risk stratum with the RF *IC *and 500 farms out of actually 10,439 available farms of the risk stratum with RF *SP *and *AM *present.

Using *cTS&bsRS*, the total minimal sample sizes required were therefore 1,241 herds for IBR and 1,750 herds for EBL on the 5%-percentile. In comparison, a *sRS *with an overall sensitivity of 99% calculated with the software Freecalc (Moffsoft™) and using the test sensitivities mentioned in Table 2 consisted of 2,259 farms to be tested for IBR and 2,243 farms to be tested for EBL.

### Analysis of cost-effectiveness

The annual serological survey for IBR and EBL in Switzerland is planned and conducted by the FVO in Berne. The samples are then collected by official veterinarians in the Regional Veterinary Offices (RVO, cantons) and sent to different diagnostic laboratories approved by the FVO for analysis. In case positive results are detected, samples are sent to the reference laboratories for IBR or EBL for confirmatory analysis. The evaluation and reporting of the results of the survey is again carried out by the FVO. The process of planning and implementation of the survey is basically identical for both *cTS&bsRS *and *sRS*. However, *cTS&bsRS *requires additional administrative effort and expenses for the annual updating of the RF per farm.

Using the values for the number of blood samples to be collected mentioned in the methods section, 25,650 individual blood samples were needed to substantiate freedom from IBR and 41,160 samples to demonstrate freedom from EBL using *cTS&bsRS*. Conventional *sRS *would require 45,180 individual blood samples to be tested for IBR and 44,860 samples for EBL.

The total costs for an IBR survey using *cTS&bsRS *amounted to 580,600 € (exchange ratio CHF/€ = 1.5), while a *sRS *cost 964,800 €. The total expenses for an EBL survey using *cTS&bsRS *added up to 880,100 €, while a *sRS *cost 955,900 € (Table 3).

**Table 3:** Comparison of costs for the annual serological survey to substantiate freedom from IBR and EBL using conventional stratified random sampling (*sRS*) and combined targeted and baseline stratified random sampling (*cTS&bsRS*) (figures based on data by S. Menéndez, Swiss Federal Veterinary Office (2008), exchange ratio CHF/€ = 1.5)

	Number of herds to be tested	Number of individual blood samples	Costs for the planning of the survey	Costs for sampling and laboratory analysis	Total costs for the full survey^5 ^
***IBR***					

**sRS**	2,259	45,180^1^	8,700 €	912,000 €	964,800 €
**cTS&bsRS**	1,241	25,650^2^	11,800 €^3^	524,500 €	580,600 €

***EBL***					

**sRS**	2,243	44,860^1^	8,700 €	903,100 €	955,900 €
**cTS&bsRS**	1,750	41,160^2^	11,800 €^3^	824,000 €	880,100 €

^1 ^The average number of animals to be tested (eq. individual samples) per farm was set at 20 for randomly selected farms.

^2 ^The average number of animals to be tested (eq. individual samples) per farm was set at 30 for farms selected targeted and at 20 for farms selected randomly (baseline sample).

^3^Additional administrative expenses for updating of "risk farms".

^4 ^The costs include farm visits, sampling labour and material, shipment/postage expenses and laboratory costs.

^5 ^The costs for the evaluation and reporting of the survey results do not differ between the two methods and are therefore not mentioned separately in the table, but are included in the total costs of the survey.

## Discussion

With the approach described in this paper, we developed a user-friendly instrument for the design of risk-based sampling programmes, providing veterinary authorities with a promising tool for future, cost-effective sampling strategies. Taking the gain in information of testing high-risk strata into account, we are able to considerably reduce the sample size. Especially for IBR, a reduction by almost half of the samples was achieved. We explain this fact by the larger number of relevant RF identified and the higher values for their respective RR compared to EBL. Another influential factor in this context is the larger number of farms available in the highest risk strata for IBR compared to EBL. The analysis of cost-effectiveness clearly revealed a financial benefit of *cTS&bsRS*, when compared to exclusive *sRS *for both IBR and EBL.

In the case of the theoretical scenario involving solely *TS *in the highest risk stratum, the reduction in sample size compared to *sRS *would be major. However, this scenario is hypothetical and based on the assumption that we have a large number of farms available in the highest risk stratum, which in reality is not the case. Nevertheless, it would be possible to conduct solely *TS *by considering all available farms in the different risk strata with the highest information gain and consequently to further decrease the necessary sample size. But the eventual geographical clustering of an entirely targeted sample due to uneven spread of risk would be a disadvantage in terms of representativeness and coverage of a survey in many regions or countries. The proposed approach of *cTS&bsRS *assures the representativeness of the survey, while at the same time taking into account the advantages of *TS*.

The stochastic scenario tree model to calculate *CSe *or *n *of the *TS *component depends on RF selected through literature review, parameterised with estimates based on expert opinion and is therefore subject to some degree of uncertainty. However, the distributions used for the RR and in consequence, consideration of conservative results on the 5%-percentile of the distribution for the *CSe *of *TS *provides a certain counterbalance for this issue. A survey based on *cTS&bsRS *guarantees an *OSe *of at least over *CSebsRS *in case the estimations for the RF and RR should have been completely inadequate. Furthermore, the percentage of *CSebsRS *on the *OSe *and therefore the degree of uncertainty can be varied and defined according to requirements. It has to be noted that correlation or dependence between RF was not considered in this study. The participants of the expert opinion survey were left free to assign any value to the RR of the evaluated RF. Although it is possible that experts intuitively considered some degree of correlation or dependence between RF, this issue was not addressed in the survey design.

Because a classical validation of a model with reliable field data is nearly impossible for rare diseases, we chose to verify the accuracy of our RF for IBR with past, well documented cases of the disease [46,47]. All of the three Swiss IBR outbreak farms from the canton of Jura in 2009 had at least one RF applying to them. One farm even had four RF. Consequently, those farms would have a high probability of being selected for a survey based on *cTS&bsRS*. For EBL however, even this attempt of validation was difficult to achieve, as only very few, poorly documented cases of leucosis actually occurred in Switzerland since the eradication of the disease.

Further surveillance components for IBR and EBL in Switzerland, such as passive clinical surveillance, slaughterhouse inspection and abortion examination, were not taken into account in this project as we aimed at analysing the legally prescribed annual serological survey only.

Additionally, we simulated and analysed the effect of varying input parameters on the SSC and directly explored the effects of several exchangeable parameters on the *OSe*. We did this using different values for *Pr_RF _*and *RR_RF _*and checking if the scenario tree model produced logical results.

The problem of testing the same farms year by year can be reduced by a yearly updating of the risk factors per farm. This is a recommended procedure anyway, as RF for the cattle farms can change over time. More importantly, the *bsRS *has a certain compensational function also in this respect. Furthermore, if a large number of farms are available in a selected high risk stratum, the farms can be selected randomly within this risk stratum, and not all farms of a certain risk stratum would have to be tested. A verification of the accuracy of and, in consequence, updating of the RF and the RR in regular time intervals (i.e. every 5 years) is also a strategy to consider.

The different approaches described in this paper are all based on whole herd testing which corresponds to the sampling framework of IBR and EBL in Switzerland to demonstrate absence of disease on the farm level. However, the model described in this paper can also be modified for diseases with increased within-herd prevalence. For such diseases, the within-herd prevalence has to be included as an additional infection node in the scenario tree model.

## Conclusions

Combined targeted and baseline stratified random sampling is a cost-effective approach for the substantiation of disease freedom and therefore has a potential to be implemented in the annual serological surveys for IBR and EBL in Switzerland. The scenario tree model described in this study can be modified, extended and further developed in order to fit other diseases and objectives for targeted surveillance in Switzerland and other countries.

## Authors' contributions

SB conducted the study and wrote the manuscript. HS participated in the design of the study, provided substantial support in the collection of data related to the risk factors, helped to supervise the work and to draft the manuscript. ME provided valuable expertise and support on the studied viruses and critically revised the manuscript. MR provided substantial support in the analysis of data related to the risk factors, helped to supervise the work and to draft the manuscript. MGD helped to draft the manuscript and co-supervised the doctoral thesis in the framework of which the manuscript was written. DCH designed the study, was the main supervisor of the work and helped to draft the manuscript. All authors read and approved the final manuscript.
